# PRPS1-mediated purine biosynthesis is critical for pluripotent stem cell survival and stemness

**DOI:** 10.18632/aging.202372

**Published:** 2021-01-20

**Authors:** Yi Yang, Lili Song, Xia Huang, Yanan Feng, Yingwen Zhang, Yanfeng Liu, Shanshan Li, Zhiyan Zhan, Liang Zheng, Haizhong Feng, Yanxin Li

**Affiliations:** 1 Key Laboratory of Pediatric Hematology and Oncology Ministry of Health, Department of Hematology and Oncology, Shanghai Children’s Medical Center, School of Medicine, Shanghai Jiao Tong University, Shanghai 200127, China; 2 State Key Laboratory of Oncogenes and Related Genes, Renji-Med X Clinical Stem Cell Research Center, Ren Ji Hospital, School of Medicine, Shanghai Jiao Tong University, Shanghai 200127, China; 3 Department of Hematology, Qilu Hospital, Shandong University, Jinan 250012, Shandong, China

**Keywords:** PRPS1/2, pluripotent stem cells, purine biosynthesis, stemness, apoptosis

## Abstract

Pluripotent stem cells (PSCs) have a unique energetic and biosynthetic metabolism compared with typically differentiated cells. However, the metabolism profiling of PSCs and its underlying mechanism are still unclear. Here, we report PSCs metabolism profiling and identify the purine synthesis enzymes, phosphoribosyl pyrophosphate synthetase 1/2 (PRPS1/2), are critical for PSCs stemness and survival. Ultra-high performance liquid chromatography/mass spectroscopy (UHPLC-MS) analysis revealed that purine synthesis intermediate metabolite levels in PSCs are higher than that in somatic cells. Ectopic expression of PRPS1/2 did not improve purine biosynthesis, drug resistance, or stemness in PSCs. However, knockout of PRPS1 caused PSCs DNA damage and apoptosis. Depletion of PRPS2 attenuated PSCs stemness and assisted PSCs differentiation. Our finding demonstrates that PRPS1/2-mediated purine biosynthesis is critical for pluripotent stem cell stemness and survival.

## INTRODUCTION

Pluripotent stem cells (PSCs) are defined by their ability to proliferate indefinitely (self-renewal) and differentiate into virtually all types of cells that comprise an organism (pluripotency) [1]. In contrast to differentiated cells, the abnormal rapidly-dividing, a shorter G1 phase of cell cycle, high ratio of nucleus to cytoplasm, and prominent nucleoli define that PSCs have a unique energetic and biosynthetic metabolism [2]. However, the metabolism profiling of PSCs is still unclear.

To facilitate rapid cell duplication, PSCs predominantly rely on glycolysis for producing ATP [3, 4]. The mitochondrial pyruvate transporter may provide a pathway to the TCA cycle for PSCs [5]. Threonine provides glycine through one-carbon metabolism for purine biosynthesis to support rapid DNA replication in mouse embryonic stem cells (mESCs), which is not required for slower proliferating fibroblasts [6]. Moreover, purine nucleotides show obvious differences between human ESCs and somatic cells [7]

Phosphoribosyl pyrophosphate synthetase 1/2 (PRPS1/2) are two homologous isoenzymes that catalyze the same biochemical reaction in both nucleotide de novo synthesis and salvage synthesis pathway [8–10]. The recombinant expression of PRPS1 protein *in vitro* exists in the form of stable hexamers which could be significantly inhibited by ADP and GDP. PRPS1 plays a vital role in proliferation and apoptosis of glioma stem cells (GSCs) [13]. Mutations of PRPS1 could cause childhood drug resistance and relapse in acute lymphoblastic leukemia (ALL) [14]. PRPS2 has lower enzymatic and allosteric activity than PRPS1, but it is sensitive to cytokine regulatory responses [11, 12]. PRPS2 is a single rate-limiting enzyme of coupling protein and nucleotide biosynthesis in Myc-driven tumorigenesis [15]. Nevertheless, many of their functions in PSCs remain to be well elucidated.

In this study, we analyzed the metabolism profiling by UHPLC-MS in PSCs and somatic cells. Then, we assessed the effects of ectopic expression or knockout (KO) of *PRPS1* or *PRPS2* on purine metabolite levels, drug sensitivity, and stemness in PSCs.

## RESULTS

### Purine synthesis intermediate metabolite levels are high in PSCs

**Figure 1:**
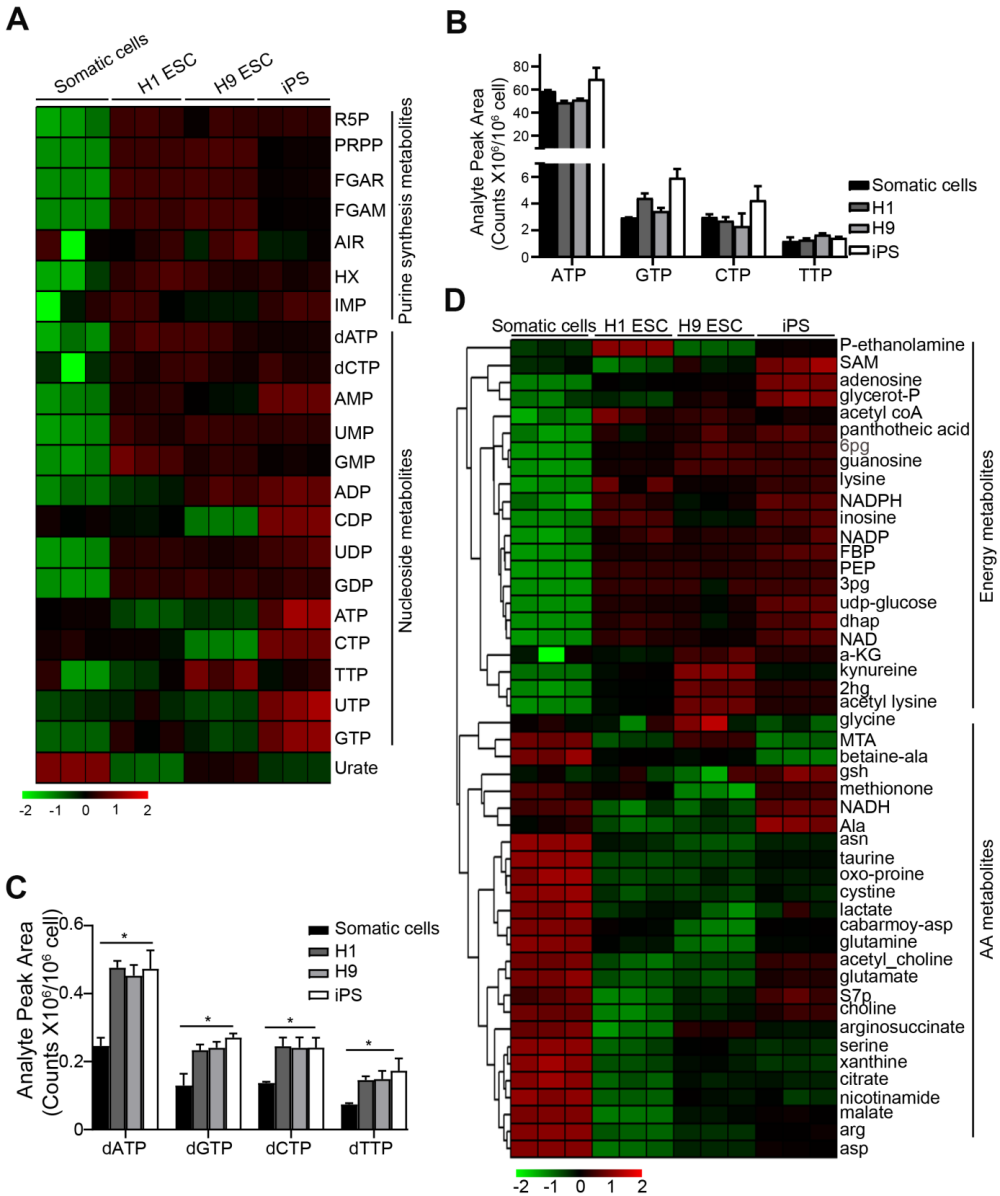
**Purine metabolite levels are high in pluripotent stem cells.** (**A**) Heatmap showing metabolomics analysis in PSCs, compared with somatic cell control using UHPLC-MS. Somatic cells, human fibroblast cells; iPS, induced pluripotency stem cells from cord blood. (**B**, **C**) Comparison of the absolute levels of NTPs (**B**) and dNTPs (**C**) in PSCs and somatic cells by analyzing analyte peak area (counts x 10^6^/10^6^ cells) from (**A**). (**D**) Heatmap showing the metabolites except for purine synthesis-related in PSCs and somatic cells. In (**C**) data are expressed as the mean ± SD. *P <0.05, by One-way ANOVA. Data are representative of three independent experiments with similar results.

To detect the metabolism profiling of PSCs, we performed metabolite analysis by UHPLC-MS in H1, H9 ESC, and iPS (induced pluripotency stem cells from cord blood) as we previously described [16]. Normal human fibroblasts (HF) cells were used as a somatic cell control. Compared to the control somatic cells, PSCs had a higher level of purine synthesis intermediate metabolite, like ribose 5-phosphate (R5P), phosphoribosyl diphosphate (PRPP), phosphoribosyl-N-formylglycineamide (FGAR), 5'-Phosphoribosylformylglycinamidine (FGAM), hypoxanthine (HX) hypoxanthine nucleotide (IMP), dATP, and dCTP [17] (Figure 1A). The level of dNTP (dATP, dGTP, dCTP, dTTP) was also higher in PSCs compared to the control, whereas the level of nucleoside triphosphates (NTPs) (ATP, CTP, TTP, UTP, GTP) had no difference between PSCs and the control cells (Figure 1A–1C). This result suggests that the purine synthesis is more important for PSCs.

Bioenergetic homeostasis and amino acids are important for rapid growth of PSCs [18]. Compared with the control somatic cells, we further found that the bioenergetic homeostasis was higher in PSCs, but the levels of amino acids were lower (Figure 1D), suggesting that there are different bioenergetic homeostasis and amino acid metabolism between somatic cells and PSCs.

### Ectopic expression of PRPS1 or PRPS2 has no effects on cell metabolism and drug resistance in PSCs

**Figure 2:**
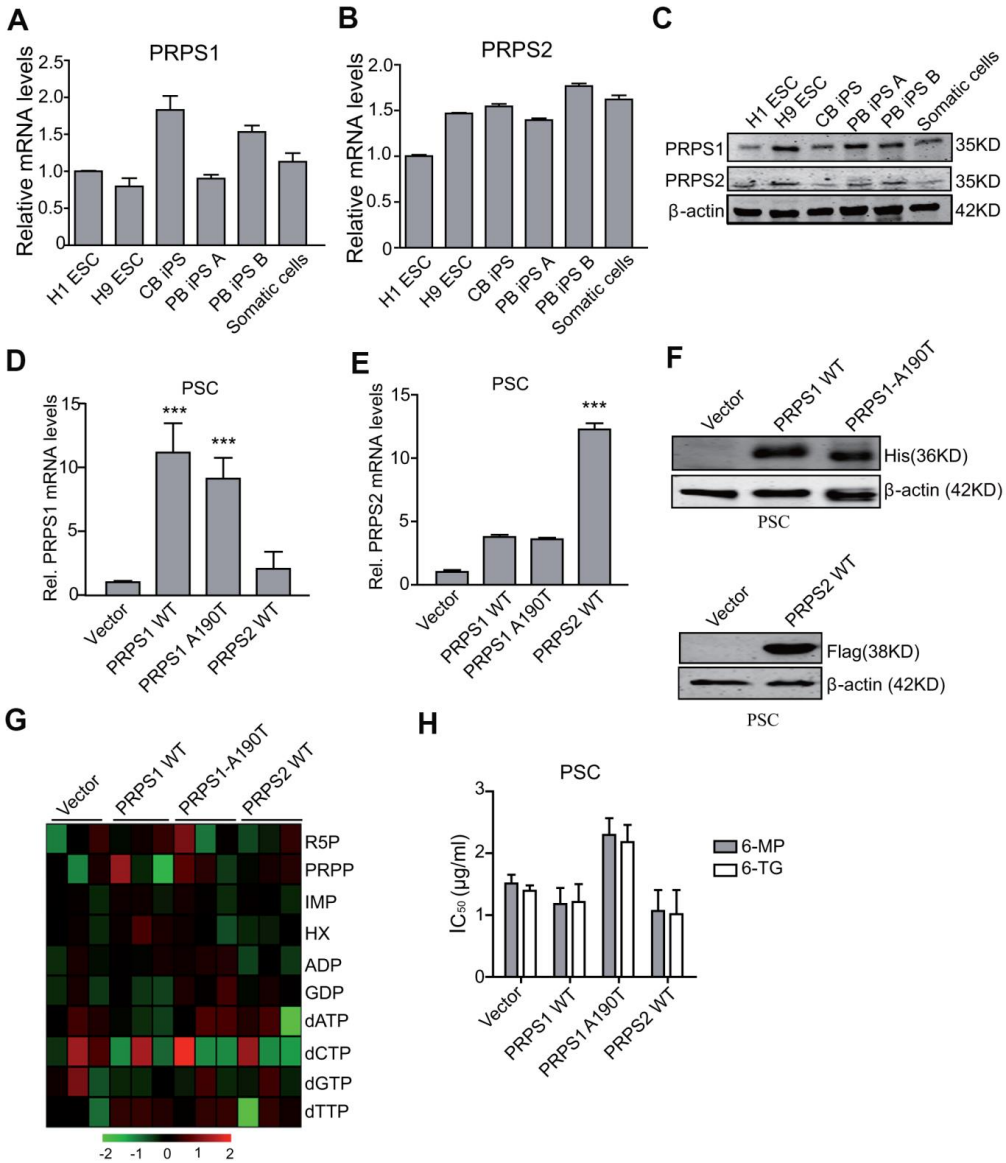
**Ectopic expression of PRPS1 or PRPS2 has no effects on cell metabolism and drug resistance in PSCs.** (**A**, **B**) qRT-PCR analysis of *PRPS1* (**A**) and *PRPS2* (**B**) mRNA levels in various PSCs and somatic cells. CB iPS, induced pluripotency stem cell derived from cord blood; PB iPS A or B, induced pluripotency stem cell derived from peripheral blood of two different healthy human. (**C**) WB of endogenous expression of PRPS1 and PRPS2 in PSCs and somatic cells from (**A**, **B**). (**D**, **E**) qRT-PCR analysis of overexpression of PRPS1 wild type (WT), WT PRPS2, or PRPS1 A190T mutant in PSCs (CB iPS cells). (**F**) WB of overexpression of PRPS1, PRPS2 or PRPS1 A190T mutant in PSCs from (**D**). (**G**) Heatmap showing the metabolomics in the PSCs from (**D**). (**H**) Effect of 6-MP or 6-TG treatment on PSCs viability. ***P< 0.001. In (**D**, **E** and **H**), data are expressed as the mean ± SD. *P <0.05, ***P <0.001, by two-tailed *t*-test. Data are representative of three independent experiments with similar results.

Since PRPS1 and PRPS2 are two crucial enzymes in the de novo and salvage synthesis of purines [8, 9, 11], we assessed whether PRPS1 and PRPS2 were important for PSCs metabolism and drug resistance. We tested the expression of PRPS1 and PRPS2 in PSCs and somatic cells by qRT-PCR and WB. As shown in Figure 2A–2C, there was no significant difference for mRNA and protein expression of PRPS1 and PRPS2 between PSCs and the control somatic cells. Any significant changes were also not observed for the other purine synthesis enzymes, PPAT, GART, HGPRT, and APRT in PSCs compared with the control somatic cells (Supplementary Figure 1).

PRPS1 wild type (WT) or the A190T mutant can not only increase purine synthesis intermediate metabolites but also lead to thiopurine 6-mercaptopurine (6-MP) and 6-Thioguanine (6-TG) resistance in relapsed childhood ALL [14]. Moreover, we recently reported that the ability of the drug resistance to thiopurine of PRPS1 depends on cell nucleotide metabolic levels [19]. Thus, we assessed whether ectopic expression of PRPS1 or PRPS2 impaired drug resistance in PSCs. We first generated stable PSCs with overexpression of PRPS1 or PRPS2 using lentivirus-mediate infection. Overexpression of His-tagged PRPS1 and Flag-tagged PRPS2 was verified by RT-PCR and WB analyses (Figure 2D–2F). Although overexpression of PRPS1 A190T reduces some sensitivity to nucleotide metabolic inhibitors (6-MP or 6-TG) in PSC with some effects on related metabolites (R5P/PRPP/dATP), compared to the empty vector control, overexpression of PRPS1, PRPS2 or PRPS1 A190T had no marked effects on cell purine and nucleotide metabolism (Figure 2G), and consistent with previous reports [14, 19], these changes did not affect cellular sensitivity to 6-MP and 6-TG in PSCs significantly (Figure 2H). These data demonstrate that PSCs already have a high level of purine metabolism, thereby PRPS1/PRPS2 overexpression or aberrant activation does not enhance purine metabolism and drug resistance again in PSCs.

### PRPS1/PRPS2 overexpression has no effects on PSCs stemness

**Figure 3:**
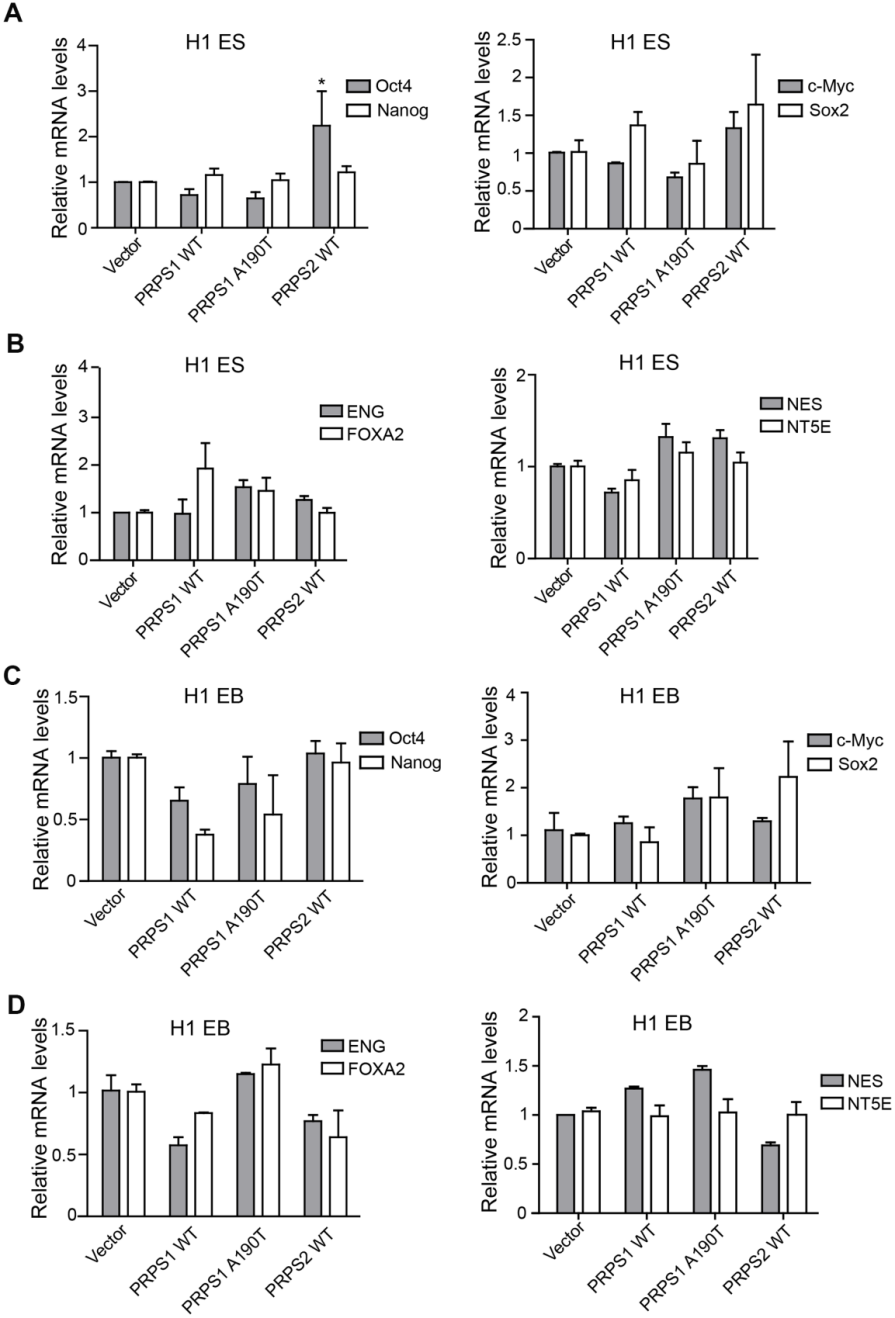
**PRPS1/PRPS2 overexpression has no effect on PSCs stemness.** (**A, C**) qRT-PCR analysis of expression levels of pluripotency genes, *Oct4*, *Nanog*, *c-Myc*, and *Sox2,* in PSCs (CB iPSCs) (**A**) and EB (embryoid bodies) (**C**). *P< 0.05. (**B**, **D**) qRT-PCR analysis of expression levels of triploblastic genes, *ENG*, *FOXA2*, *NES*, and *NT5E,* in PSCs (**B**) and EB (embryoid bodies) (**D**) Data are representative of three independent experiments with similar results.

High expression of PRPS1/2 is a key driver for cancer stem cell high activity, including maintaining stem cell self-renewal, proliferation and even catalyzing tumorigenesis [15]. Thus, we assessed the effects of PRPS1/PRPS2 overexpression on PSCs stemness. We analyzed the expression levels of four pluripotency genes (Oct4, c-Myc, Nanog, and Sox2) and four triploblastic genes (NT5E, NES, NEG, and FOXA2) at PSCs and EB stages using qRT-PCR. Among four pluripotency genes, only Oct4 expression level was markedly high in PSCs with PRPS2 overexpression compared to other PSCs indicated (Figure 3A). Overexpression of WT PRPS1 or the A190T mutant had no effects on pluripotency and triploblastic genes expression (Figure 3A, 3B). Overexpression of WT PRPS1, PRPS1 A190T mutant, or WT PRPS2 did not impair pluripotency and triploblastic genes expression in the stage of EB (Figure 3C, 3D). These data demonstrate that overexpression of PRPS1 or PRPS2 has no effect on PSCs stemness.

### PRPS2 mediates PSCs stemness

**Figure 4:**
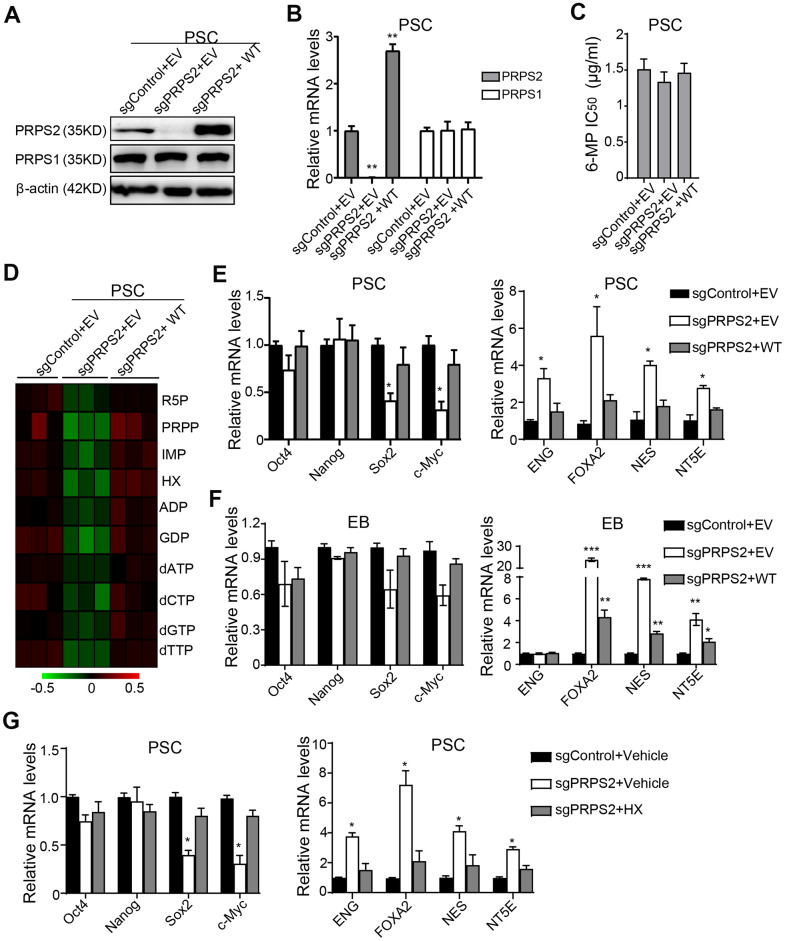
**PRPS2 mediates PSCs stemness by PRPS2-regulated purine metabolism.** (**A**, **B**) WB (**A**) and qRT-PCR (**B**) analysis of effects of PRPS2 KO or re-expression on PRPS1 expression in PSCs. WT, sgPRPS2 resistant WT PRPS2. (**C**) Effect of 6-MP treatment on *PRPS2* KO or re-expression PSCs’ viabilities. (**D**) Heatmap showing metabolomics analysis in PSCs from (**A**). (**E**, **F**) qRT-PCR analysis of effects of *PRPS2* KO or re-expression on the expression of pluripotency genes or triploblastic genes in PSCs (**E**) and EBs (embryoid bodies) (**F**). (**G**) qRT-PCR analysis of effects of *PRPS2* on the expression of pluripotency genes and triploblastic genes in response to HX treatment. In (**B**, **E**, **F** and **G**), data are expressed as the mean ± SD. *P <0.05, **P <0.01, ***P <0.001, by two-tailed *t*-test. Data are representative of three independent experiments with similar results.

Since PRPS1/2 is dispensable for normal physiology and cellular function in mature cells [15], we investigated whether *PRPS2* KO affected PSCs purine metabolism and stemness. *PRPS2* KO PSCs cell lines were successfully established using the sgRNA technology and identified by WB (Figure 4A) and qRT-PCR (Figure 4B) assays. Compared to the vector control, *PRPS2* KO or re-expression of sgRNA-resistant WT PRPS2 had little or no influence on PRPS1 expression (Figure 4A, 4B). *PRPS2* KO or re-expression also had no effect on 6-MP resistance in PSCs (Figure 4C). We further analyzed the metabolism by UHPLC-MS and found that although there was no significant difference among control, PRPS2 KO and re-expression, *PRPS2* KO led to a decrease in purine synthesis intermediate metabolites. In contrast, *PRPS2* re-expression rescued *PRPS2* KO-inhibited purine metabolites (Figure 4D). These results support that PRPS2 may play a minor role in purine metabolism in PSCs.

Next, we assessed the pluripotency and triploblastic genes expression at PSCs and EB stages. As shown in Figure 4E, expression levels of pluripotent gene, Sox2 and c-Myc, were significantly decreased in *PRPS2* KO PSCs, whereas expression levels of triploblastic gene, ENG, FOXA2, NES, and NT5E, were generally increased. In contrast, *PRPS2* re-expression rescued *PRPS2* KO-inhibited Sox2 and c-Myc expression and *PRPS2* KO-increased triploblastic gene expression (Figure 4E). At EB stage, the expression levels of pluripotent genes had no significant changes in *PRPS2* KO cells, whereas expression levels of triploblastic genes of FOXA2, NES, and NT5E were markedly increased. *PRPS2* re-expression rescued *PRPS2* KO-increased triploblastic gene expression (Figure 4F). These data demonstrate that PRPS2 is important for PSCs.

To further determine the relationship between PRPS2-mediated stemness and purine metabolism in PSCs, we used HX, a naturally occurring purine derivative, to treat *PRPS2* KO PSCs and analyzed the expression of pluripotent genes and triploblastic genes. As shown in Figure 4G, compared to the medium treatment control, HX treatment could rescue *PRPS2* KO-decreased Sox2 and c-Myc expression and *PRPS2* KO-increased the triploblastic gene expression. These data suggest that PRPS2-regulated PSCs stemness depends on PRPS2-mediated PSCs purine metabolism.

### PRPS1 is critical for PSCs survival and stemness by controlling purine synthesis

**Figure 5:**
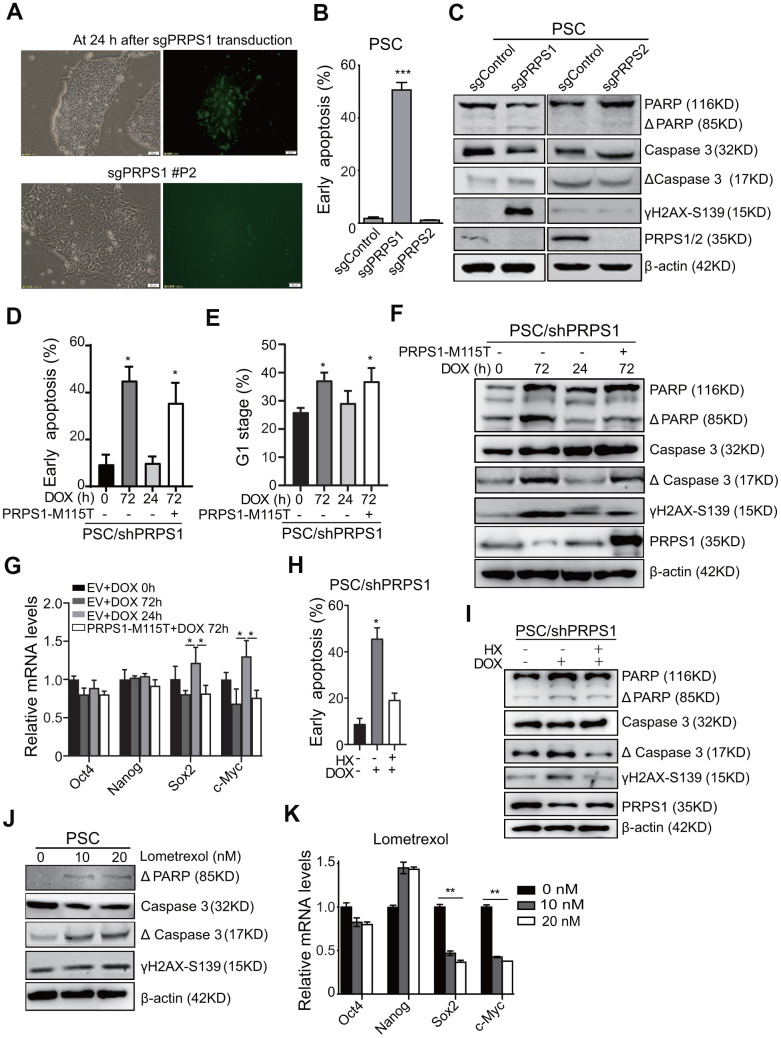
**PRPS1 is critical for PSCs survival and stemness by controlling purine synthesis.** (**A**) Representative images of cells at 24 h post sgRNA-PRPS1 lentivirus infection or at second passage (P2). Bars: 20 μm. (**B**) FACS analysis of cell apoptosis in PSCs at Day 3 after sgPRPS1 or sgPRPS2 lentivirus transduction. *** *P*< 0.001. sgControl, a control sgRNA. (**C**) WB of the expression of DNA damage and apoptosis marker proteins in the PSCs from (**B**). (**D**, **E**) FACS analysis of cell apoptosis (**D**) and cell cycle (**E**) in DOX-induced *PRPS1* KD PSCs with or without ectopic expression of PRPS1 M115T mutant. DOX was added for 0h, 72h, or 24 h to induce *PRPS1* KD. At 24 h, DOX were removed to rescue PRPS1 expression. PRPS1-M115T mutant, an enzymatically inactive PRPS1 mutant. (**F**) WB of the expression of DNA damage and apoptosis marker proteins in the PSCs from (**D**). (**G**) qRT-PCR analysis of the expression levels of pluripotency genes in the PSCs from (**D**). (**H**) FACS analysis of cell apoptosis in response to HX treatment based on *PRPS1* KD. (**I**) WB of the expression of DNA damage and apoptosis marker proteins in response to HX treatment based on *PRPS1* KD. (**J**) WB of the expression of DNA damage and apoptosis marker proteins in response to Gart inhibitor, lometrexol, treatment. (**K**) qRT-PCR analysis of the expression levels of pluripotency genes in the PSCs from (**J**). In (**B**, **D**, **E**, **G**, **H** and **K**), data are expressed as the mean ± SD. *P <0.05, ***P <0.001, by two-tailed *t*-test. Data are representative of three independent experiments with similar results.

To investigate the role of PRPS1 in PSCs stemness and purine metabolism, we transduced sgRNA-PRPS1 lentiviruses with GFP (Green Fluorescent Protein) into PSCs. As shown in Figure 5A, 24 h after sgRNA-PRPS1 transduction, the GFP positive PSCs were detected by fluorescent microscope and the infection efficiency was ~ 30%. The GFP positive PSCs, however, disappeared after the second cell passage (P2) (Figure 5A). We analyzed PSCs apoptosis by Annexin V assay at Day 3 after sgPRPS1 or sgPRPS2 lentivirus transduction and found that the apoptosis rate in PRPS1 KO PSCs was significantly higher than that in *PRPS2* KO and the vector control PSCs (Figure 5B). We also transduced the same sgRNA-PRPS1 lentiviruses into leukemia Reh cells. Results showed that *PRPS1* KO had no significant effects on Reh apoptosis at Day 3 after lentivirus transduction (Supplementary Figure 2A). We further assessed apoptosis-related protein expression in PSCs at Day 3 after sgRNA-PRPS1 lentivirus transduction and confirmed that *PRPS1* KO increased the expression of γH2AX-S319, cleavage PARP and caspase 3, whereas *PRPS2* KO had no effects on apoptosis-related protein expression (Figure 5C). These results indicate that PRPS1 is very important for PSCs survival.

To validate our observations above, we established the DOX-induced PRPS1 shRNA knockdown (KD) PSCs and selected PRPS1 shRNA#1 cell line based on PRPS1 expression (Supplementary Figure 3A). As shown in Figure 5D, compared to the un-induced control, the inducible *PRPS1* KD by 72-h DOX treatment markedly increased cell apoptosis (Figure 5D), inhibited cell cycle (Figure 5E), and promoted apoptosis-related protein expression (Figure 5F) in PSCs. Withdrawing DOX after 24-h DOX treatment successfully rescued PRPS1 expression to a certain degree and reversed *PRPS1* KD-induced cell behaviors (Figure 5D–5F), whereas ectopic expression of the PRPS1 enzymatically inactive M115T mutant did not (Figure 5D–5F). When we transduced the DOX-induced PRPS1 shRNA1 or PRPS1 sgRNA into HF cells, we did not find PRPS1 KD or KO could increase apoptosis and related protein expression (Supplementary Figure 2B, 2C). These data further support that PRPS1 is critical for PSCs survival.

We further assessed whether PRPS1-mediated purine metabolism was also important for PSCs stemness. As shown in Figure 5G, Supplementary Figure 3B, the inducible *PRPS1* KD, PRPS1 expression, or ectopic expression of PRPS1 M115T mutant had no significant effects on the expression of pluripotent and triploblastic genes compared to the empty vector control. However, *PRPS1* knockdown significantly decreased the purine synthesis intermediate metabolites, and this intermediate metabolite decease could be rescued by PRPS1 re-expression but not the PRPS1 M115T mutant (Supplementary Figure 3C). We treated *PRPS1* KD PSCs with HX and found that HX treatment rescued *PRPS1* KD-induced PSCs apoptosis and the apoptosis-related protein expression (Figure 5H, 5I). Treatment of lometrexol, a Gart inhibitor in purine synthesis [14], increased apoptosis and DNA damage in PSCs (Figure 5J). Additionally, lometrexol treatment inhibited the expression of Sox2 and c-Myc in PSCs (Figure 5K). These results demonstrate that PRPS1 is critical for PSCs survival and stemness through controlling purine synthesis.

## DISCUSSION

In this study, we show the metabolism profiling of PSCs and reveal that purine synthesis intermediate metabolite levels in PSCs are higher than that in somatic cells. Moreover, we identify that PRPS1 and PRPS2 play different roles in purine synthesis, stemness, and survival in PSCs.

PRPS1 and PRPS2 are two crucial enzymes in purine metabolism [11, 14], and their expression levels are highly tissue-specific [20]. For example, PRPS1 level is high in the brain, while PRPS2 prevails in the lung and spleen. Both genes were highly expressed in lymphocytes [20]. In this study, we show that PSCs expressed high levels of PRPS1 and PRPS2 to sustain a high level of purine metabolism. Compared to somatic cells, the expression levels of PRPS1 and PRPS2 in PSCs were similar, whereas PSCs showed a relatively higher level of purine metabolism. Moreover, overexpression of PRPS1 and PRPS2 did not affect purine synthesis, drug resistance, and stemness. High purine metabolism in PSCs may be an attribute to achieve rapid cell proliferation [3]. The insight of this unique metabolism warrants further investigation.

Purine is synthesized through de novo and salvage pathways [8], and it is important for PSCs. However, knockout of the limiting enzyme HGPRT in the salvage pathway does not impair any function of ESCs cells [17]. In brain tumor initiating cells, combined metabolomics and genomic analyses reveal specific upregulation of de novo purine synthesis through PRPS1[21]. PRPS1 promotes pentose phosphate pathway-dependent de novo nucleic acid synthesis and hepatocellular carcinoma formation [22]. Abnormal metabolism of purine induced by PRPS1 mutations could cause severe disease, and some lose-of-function mutations of PRPS1 even cause human embryonic lethality [23]. Our data suggest that the de novo purine synthesis mediated by PRPS1 plays a critical role in PSCs. PRPS1 KO and Gart inhibitor treatment impair purine synthesis through de novo pathway and increase DNA damage and apoptosis in PSCs but attenuate PSCs stemness.

Although PRPS1 and PRPS2 are two isoforms of the human PRPS family and have 95% amino acid sequence identity [11, 14], their functions are different. PRPS1 is required for purine metabolism in glioma [21], colorectal cancer [24], and hepatocellular carcinoma formation [22]. PRPS2 couples protein and nucleotide biosynthesis in Myc-driven tumorigenesis [15]. In this study, we find that PRPS2 plays minor roles in purine synthesis, stemness, and survival in PSCs but inhibits PSCs differentiation.

Taken together, our study discovers the metabolism profiling of PSCs, which is in line with previous reports. Moreover, we identify that PRPS1 is critical for cell purine metabolism, survival, and stemness in PSCs. Our finding will contribute to a comprehensive understanding of PSCs metabolism and PRPS1/2 functions in PSCs stemness.

## MATERIALS AND METHODS

### Cell culture

HEK-293T cells and human fibroblasts (HF) were cultured in Dulbecco’s modified Eagle’s medium (DMEM, Gibco) supplemented with 10% fetal bovine serum (FBS, Life Technologies) and 1% Penicillin-Streptomycin (NCMBIO). Leukemia Reh cells were cultured in RPMI Medium 1640 basic (Gibco) supplemented with 10% FBS (Life Technologies). All cells were incubated at 37° C in 5% CO_2_. Pluripotent stem cells: H1 and H9 embryonic stem cell (ESCs) were bought from ATCC, induced pluripotent stem cells were derived as we previous described [16]. They were maintained in a feeder-free culture system. Briefly, we precoated the well plates with Matrigel (BD Biosciences), and then seeded the PSCs and cultured them in E8 medium.

### PSCs differentiation into embryoid bodies (EBs)

PSCs were differentiated into embryoid bodies (EBs) as we previous described [25]. Briefly, 3000 PSCs cells were plated in differentiation medium (DMEM/F12, Gibco) supplemented with 15% Knockout serum replacement (KSR), 1x non-essential amino acids, 2 mM Glutamine, and 0.1 mM β-mercaptoethanol (specialty media, Chemicon) without bFGF, poly (2-hydroxyethyl methacrylate)-coated 96-well plates that promoted the formation of floating cell aggregates. EBs were collected for RNA extraction at day 10. All of cell lines were tested for mycoplasma contamination every week.

### Plasmids, lentivirus production, and infection

Human PRPS1 and PRPS2 coding regions were cloned into pGV287 Vector (GeneChem, Shanghai). The A190T point mutant was constructed using site-directed mutagenesis and confirmed by DNA sequencing. LentiCRISPR v2 was a gift from Feng Zhang (Addgene plasmid #52961) [26]. sgRNAS were designed following the protocol of Zhang laboratory (http://crispr.mit.edu) (PRPS1: 5’-TCAGAAAATTGCTGACCGCC-3’, PRPS2: 5’-GTTCAGCGGCAGCTCGCATC-3’). The lentiviral doxycycline-inducible GFP-IRES-shRNA FH1tUTG construct [27] was a gift from Dr. Marco Herold (Walter and Eliza Hall Institute of Medical Research, Melbourne, Australia). PRPS1 shRNA #1 and PRPS1 shRNA #2 target sequences were 5’-CAGGAGGACAAGATGAAGCA-3’, 5’-GCTTGTTGCAAATATGCTA-3’ respectively. Sense and antisense control shRNA, PRPS1 shRNA #1 and PRPS1 shRNA #2 oligoes were cloned into the DOX-inducible GFP-IRES-shRNA FH1tUTG construct. The lentiviral constructs were transfected into HEK-293T cells with packaging plasmids pSPAX2 and pMD2G using the calcium phosphate method to produce replication-defective virus. The supernatant was harvested 48 h later and concentrated by 100 kDa column (Amicon purification system, MILLIPORE). PSCs were virally transduced with supplemented with 4 μg/ml polybrene (Sigma). The medium was changed 24 h after infection, and GFP-positive cells were sorted using MoFlo XDP (Beckman Coulter, CA, USA).

### Cell viability

Cell viability was determined by using Cell Titer-Glo Luminescent kit (Promega) according to the manufacturer’s instructions. Cells were seeded in 96-well plates at 10,000 per well and treated with drugs of different serial dilutions for 72 h. Then, the Cell Titer-Glo Reagents (50 UI) were added to each well and mixed for 10 min before the luminescent signal was measured using a microplate reader (Biotek, VT, USA).

### Cell cycle

These assays were performed using flow cytometry as we previously described [28] Briefly, PSCs were fixed in cold 70% ethyl alcohol for 30 min at 4° C, and then were treated with 100μg/ml Ribonuclease. At the same time, 200μl cell suspension were incubated with 50μg/ml propidium iodide for 1 h at room temperature. Flow cytometry analysis was performed.

### Western blotting (WB)

Cells were lysed and standard WB was performed with antibodies against His-Tag (D3I10, Cell Signaling Technology), Flag-Tag (M1403-2, HuaBio), β-actin (I-19, Santa Cruz Biotechnology), PRPS2 (NBP1-56666, Novus Biologicals), PRPS1 (sc-376440, Santa Cruz Biotechnology), PAPP (#46D11, Cell Signaling Technology), cleaved PAPP (#46D11, Cell Signaling Technology), Caspase 3 (#9668, Cell Signaling Technology), cleaved caspase 3 (#9668, Cell Signaling Technology), γH2AX-S139 (#3522-1, Epitomics) using the Odyssey system (LI-COR Biosciences) [29].

### qRT-PCR analysis

Somatic cells, PSCs and EBs were collected as previously described [25]. Then, total RNAs were extracted using the RNeasy plus kit (Qiagen) in accordance with manufacturer’s protocol. Real-time PCR was performed using the SYBR Green PCR Master Mix (Applied Biosystems, Foster City, CA, USA) on a 7500 Fast Real-Time PCR System (Applied Biosystems). Primers were listed in Supplementary Table 1.

### Metabolite flux assays

Cells were harvested at a density of 5 x 10^5^/ml, pelleted and quenched in cold 80% methanol, centrifuged at 12,000 rpm for 10 min, and the supernatant was applied for metabolite analysis by ultra-high performance liquid chromatography/mass spectroscopy (UHPLC-MS; Thermo Scientific, UPLC with Q Exactive Plus mass spectrometer) as described previously [14, 19].

### Flow cytometry analysis (FACS) of apoptosis

Various PSCs (2 x 10^5^) were collected at Day 3 after transduction. The cells were stained with the FITC Annexin V Apoptosis Detection Kit II (R&D, Minneapolis, MN) and propidium iodide (Sigma, St Louis, MO, USA) according to the manufacturer’s recommendations and analyzed by flow cytometry with vector as controls.

### Statistical analysis

All data represented the mean ± SD of three independent experiments/samples unless specifically indicated. Two-tailed Student *t*-test was performed, and *P* < 0.05 was considered statistically significant.

## Supplementary Material

Supplementary Figures

Supplementary Table 1
