# Supplementary material for: PRPS1-mediated purine biosynthesis is critical for pluripotent stem cell survival and stemness
Source: Aging (Albany NY). 2021 Jan 20;13(3):4063–78. doi: 10.18632/aging.202372 (PMC7906169; doi:10.18632/aging.202372)
Supplement: Supplementary Figures [file aging-13-202372-s001.pdf]

SUPPLEMENTARY FIGURES

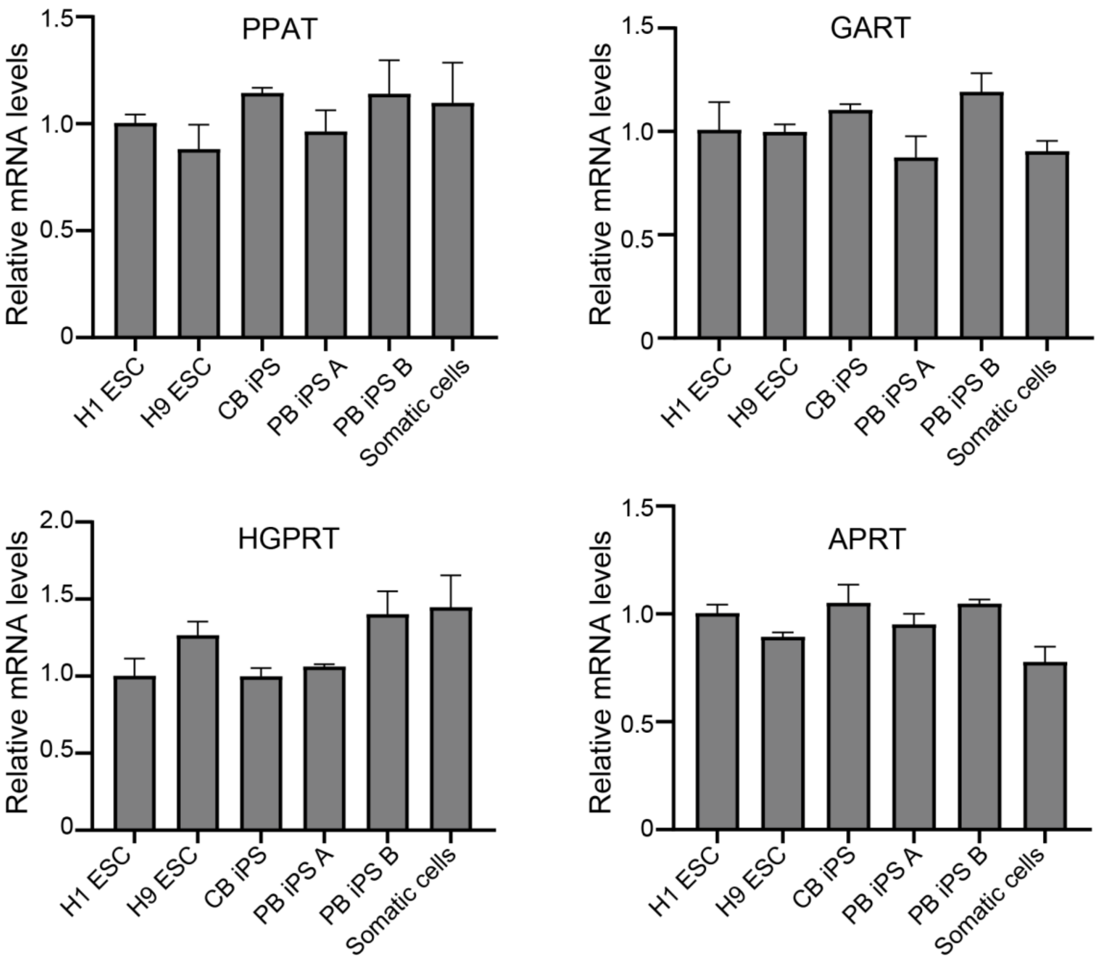

Supplementary Figure 1. qRT-PCR analysis of the expression levels of *PRAT*, *GART*, *HGPRT*, and *APRT* mRNA in various PSCs lines. CB iPS, induced pluripotency stem cell derived from cord blood; PB iPS A, induced pluripotency stem cell derived from peripheral blood of a normal human A; Somatic cells, human fibroblast cells (HF).

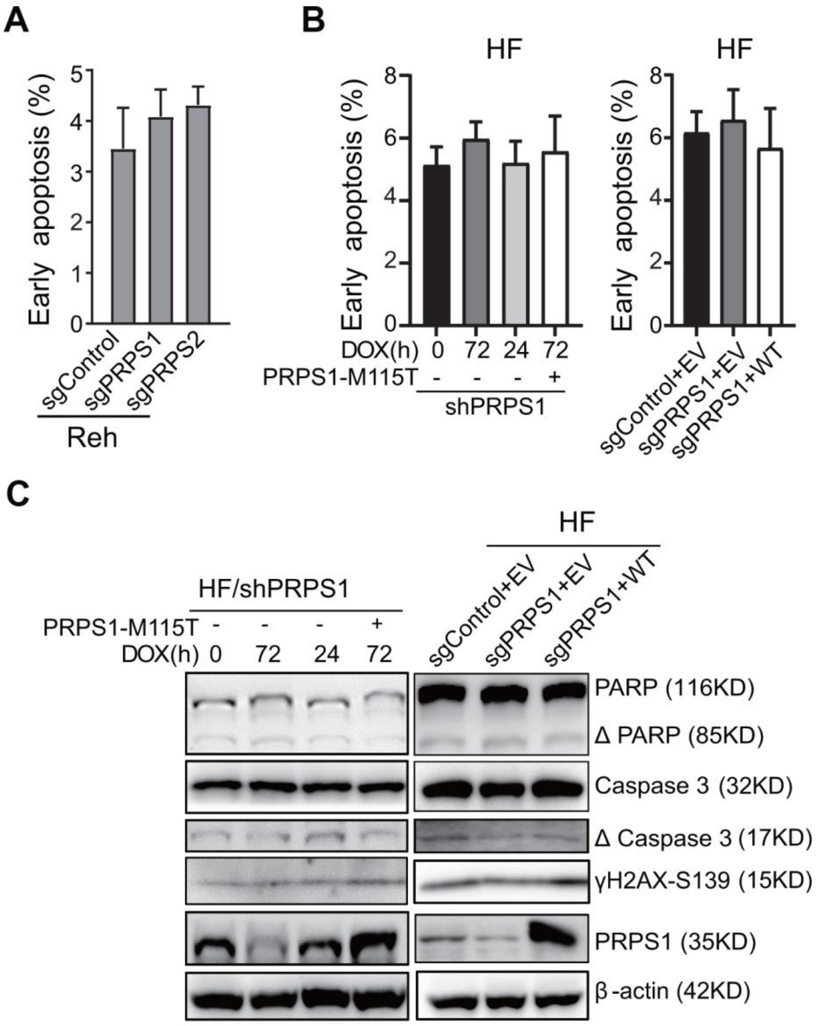

Supplementary Figure 2. FACS of cell apoptosis and WB of effects of *PRPS1* knockdown (KD), *PRPS1* knockout (KO), or *PRPS2* KO on the expression of apoptosis and DNA damage marker proteins. (**A**) FACS of cell apoptosis in Reh at Day 3 after sgRNA-PRPS1 and sgRNA-PRPS2 lentivirus infection. (**B**) FACS of cell apoptosis in DOX-induced *PRPS1* KD or KO HF cells. Cells were collected at Day 3 after treated with or without Dox. PRPS1-M115T, a PRPS1 enzymatically inactive mutant. WT, a sgPRPS1 resistant WT PRPS1. (**C**) WB of the expression of DNA damage and apoptosis marker proteins in the cells from (**B**).

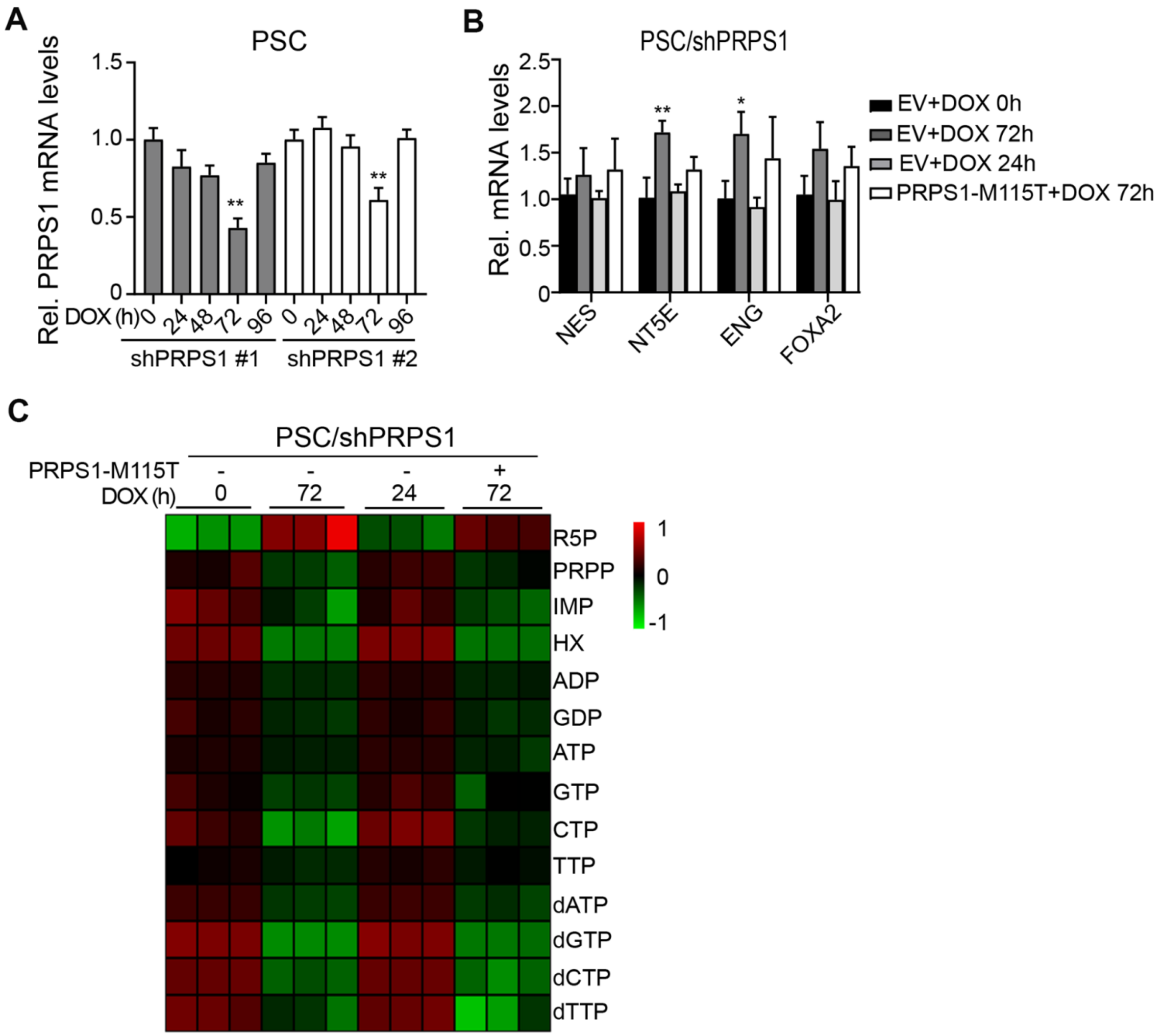

Supplementary Figure 3. qRT-PCR analysis of the effects of PRPS1 WT or the M115T mutant on the expression of triploblastic genes and purine metabolite. (**A**) qRT-PCR analysis of the expression levels of *PRPS1* in Dox-induced shPRPS1 PSCs. (**B**) qRT-PCR analysis of the expression levels of *NES*, *NT5E*, *ENG*, and *FOXA2* in the cells from (**A**). (**C**) Heatmap showing metabolomics analysis in the cells from (**A**).
