# Supplementary material for: PRPS1-mediated purine biosynthesis is critical for pluripotent stem cell survival and stemness
Source: Aging (Albany NY). 2021 Jan 20;13(3):4063–78. doi: 10.18632/aging.202372 (PMC7906169; doi:10.18632/aging.202372)
Supplement: Supplementary Table 1 [file aging-13-202372-s002.pdf]

## SUPPLEMENTARY TABLE

**Supplementary Table 1. Primers for qRT-PCR.**

| Genes | Primer Sequence (5'—3') | |
|---|---|---|
| | Forward | Reverse |
| Oct4 | TGTACTCCTCGGTCCCTTTC | TCCAGGTTTTCTTTCCCTAGC |
| Sox2 | GCTAGTCTCCAAGCGACGAA | GCAAGAAGCCTCTCCTTGAA |
| Nanog | CAGTCTGGACACTGGCTGAA | CTCGCTGATTAGGCTCCAAC |
| C-MYC | GAATGTCAAGAGGCGAACACA | CGTCGTTTCCGCAACAAG |
| ENG | TCCTCCCAAGGACACTTGTA | GCGCAACAAGCTCTTTCTTTAG |
| FOXA2 | GGCCCATTATGAACTCCTCTT | CTTGCTCTCTCACTTGTCCTC |
| NES | CACTCCAGTTTAGAGGCTAAGG | CCCTCTATGGCTGTTTCTTTCT |
| NT5E | GGAGATGGGTTCCAGATGATAAA | CGACCTTCAACTGCTGGATAA |
| PRPS1 | GATCTATTTGGCCTCTCAAA | CACACAGGTACACACACTTTATT |
| PRPS2 | TGAAGGACCGTGTGGCCAT | TAGCTGGTCCAGAGAAGATCCCAT |
| PPAT | GATGGGAGTTCGGTGCCAA | CAACGAAGGGCTGACAATTTTC |
| GART | CTGGAAAAGGGGTGATTGTTGC | TCAGTGAAACACAGACACGAC |
| HGPRT | CCTGGCGTCGTGATTAGTGAT | AGACGTTCAGTCCTGTCCATAA |
| APRT | GGCCGCATCGACTACATCG | CTCAGCCTTCCCGTACTCC |
